# Supplementary material for: ADRA2A promotes the classical/progenitor subtype and reduces disease aggressiveness of pancreatic cancer
Source: bioRxiv. 2024 Mar 13:2024.03.12.584316. Preprint. [Version 1] doi: 10.1101/2024.03.12.584316 (PMC11188071; doi:10.1101/2024.03.12.584316)
Supplement: Supplement 4 [file NIHPP2024.03.12.584316v1-supplement-4.pdf]

bioRxiv preprint doi: https://doi.org/10.1101/2024.03.12.584316; this version posted March 13, 2024. The copyright holder for this preprint (which was not certified by peer review) is the author/funder. This article is a US Government work. It is not subject to copyright under 17 USC 105 and is also made available for use under a CC0 license.

Supplementary figure legends

Supplementary Figure 1. ADRA2A expression and analysis by molecular subtypes in PDAC. (A) *ADRA2A* mRNA expression by molecular subtypes in the NCI-UMD-German cohort. (B) Gene Set Enrichment Analysis (GSEA) suggests that elevated ADRA2A expression leads to the downregulation of the predefined basal-like gene expression profile (29). (C and D) Endogenous levels of ADRA2A mRNA and protein in various human PDAC cell lines, revealing consistent suppression of ADRA2A expression across all examined PDAC cell lines. (E-H) Confirmation of *ADRA2A* transgene overexpression at the mRNA (qPCR) and protein levels in CFPAC-1 and Panc 10.05 cells. Scale bar is 50 μm. Data represent mean ± SD with ANOVA. ****$p \leq 0.0001$. N.D.; not detected.

bioRxiv preprint doi: https://doi.org/10.1101/2024.03.12.584316; this version posted March 13, 2024. The copyright holder for this preprint (which was not certified by peer review) is the author/funder. This article is a US Government work. It is not subject to copyright under 17 USC 105 and is also made available for use under a CC0 license.

Supplementary Figure 2. Downregulation of amino acid metabolism in PDAC cells with a MYC inhibitor. (A and B) Metabolome analysis covering 116 cancer-related metabolites in BxPC-3 cells with a MYC inhibitor (10058-F4, 100 μM). In BxPC-3 cells with a MYC inhibitor, 48 metabolites were significantly downregulated when compared with solvent control cells ($p < 0.05$). The graphs illustrate the decrease in amino acid metabolism. Data are presented as mean ± SD. (C) Pathway enrichment scores using MetaboAnalyst 5.0 (25) with the significantly decreased 48 input metabolites, indicating that amino acid metabolism were downregulated in BxPC-3 cells with a MYC inhibitor. Data represent mean ± SD of four replicates. ****$p \leq 0.0001$ by unpaired two-tailed Student's t-test.

(which was not certified by peer review) is the author/funder. This article is a US Government work. It is not subject to copyright under 17 U
105 and is also made available for use under a CC0 license.

Supplementary Figure 1

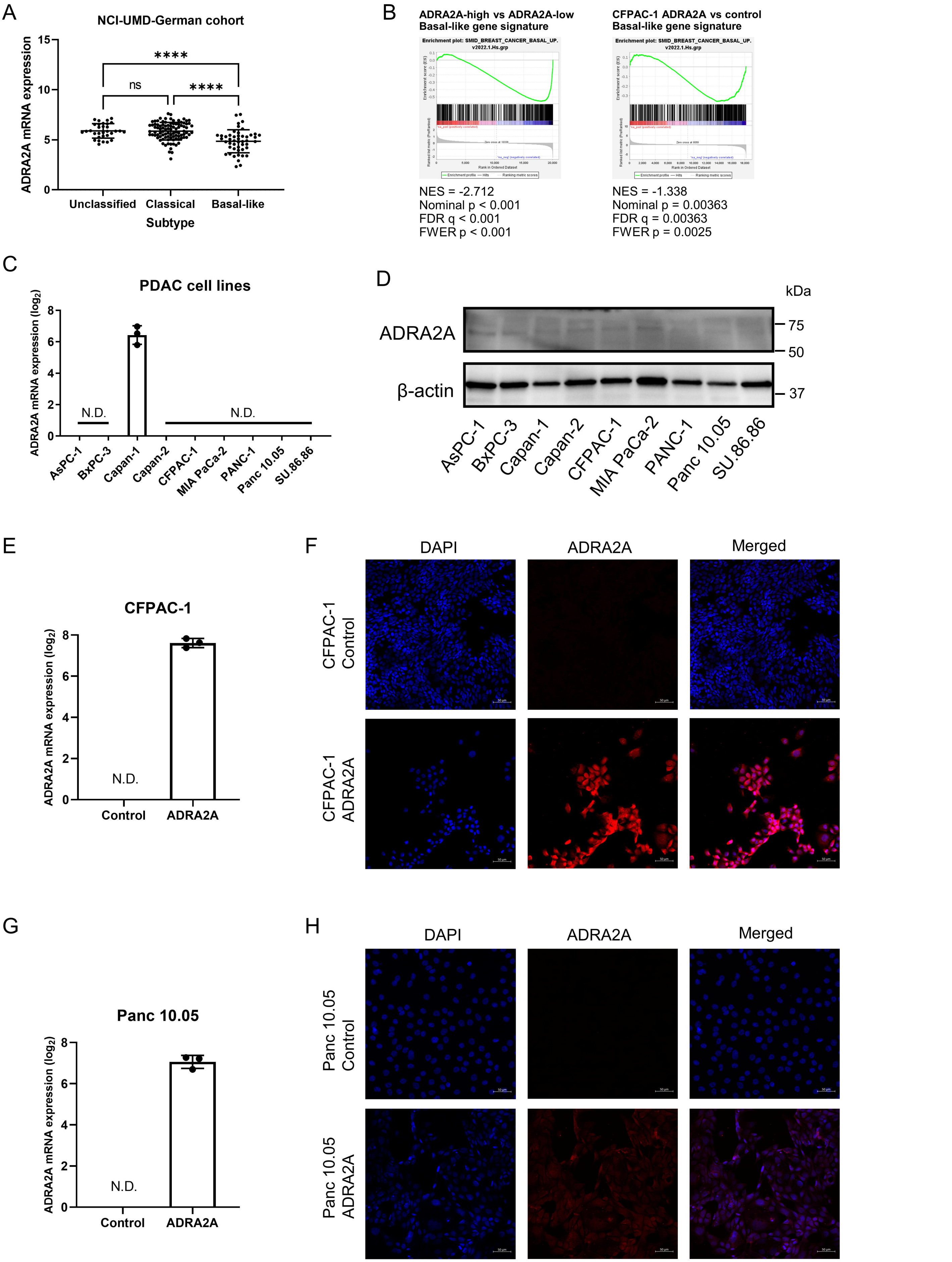

Supplementary Figure 2

A

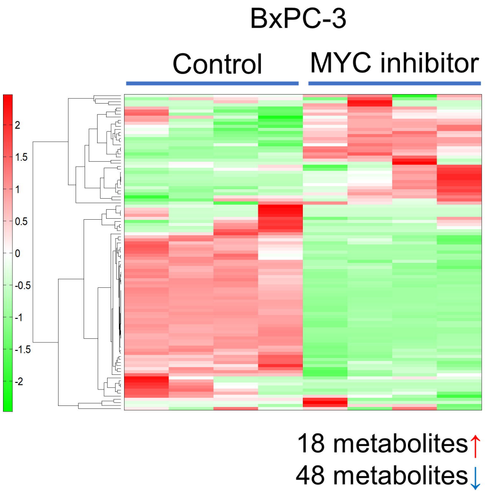

B

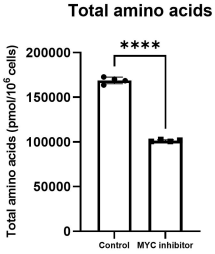

C

Pathways	*P* value
Alanine, aspartate and glutamate metabolism	5.38E-09
Arginine biosynthesis	8.31E-08
Arginine and proline metabolism	8.54E-08
D-Amino acid metabolism	3.92E-06
Valine, leucine and isoleucine biosynthesis	6.80E-05
Citrate cycle (TCA cycle)	3.49E-04
beta-Alanine metabolism	4.46E-04
Glutathione metabolism	1.81E-03
Glyoxylate and dicarboxylate metabolism	2.90E-03
Pantothenate and CoA biosynthesis	3.50E-03
Phenylalanine, tyrosine and tryptophan biosynthesis	6.19E-03
Cysteine and methionine metabolism	2.15E-02
Phenylalanine metabolism	2.65E-02
Valine, leucine and isoleucine degradation	3.74E-02
Pentose phosphate pathway	3.81E-02
Pyruvate metabolism	3.81E-02
